# Supplementary material for: Translation of the Weight-Related Behaviours Questionnaire into a Short-Form Psychosocial Assessment Tool for the Detection of Women at Risk of Excessive Gestational Weight Gain
Source: Int J Environ Res Public Health. 2021 Sep 9;18(18):9522. doi: 10.3390/ijerph18189522 (PMC8472452; doi:10.3390/ijerph18189522)
Supplement: Supplementary file 1 [file ijerph-18-09522-s001.zip › ijerph-1322328-supplementary.pdf]

1. Weight Locus of Control

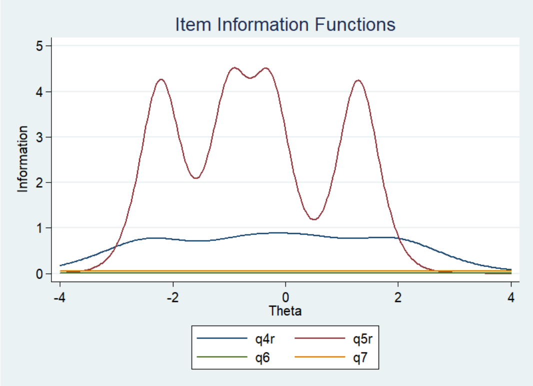

2. Self efficacy

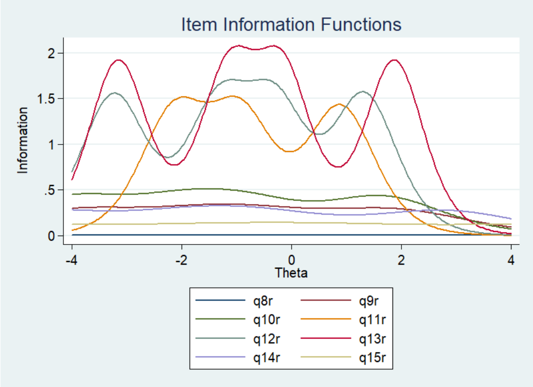

3. Attitudes towards weight gain

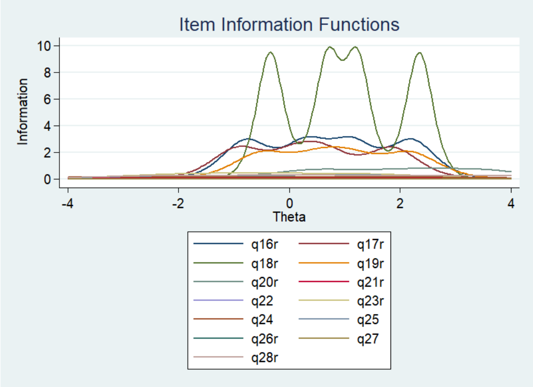

4. Body Image

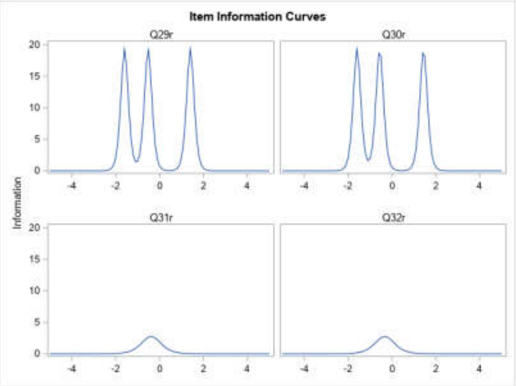

5. Feelings about the motherhood role

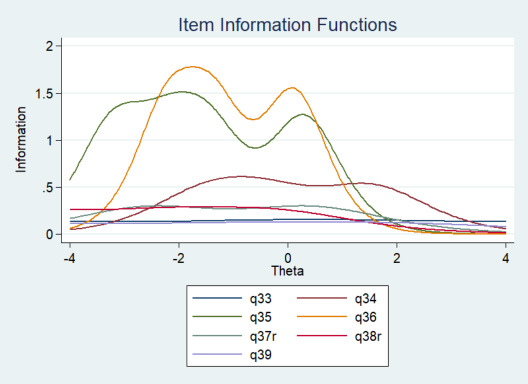

6. Career oreintation

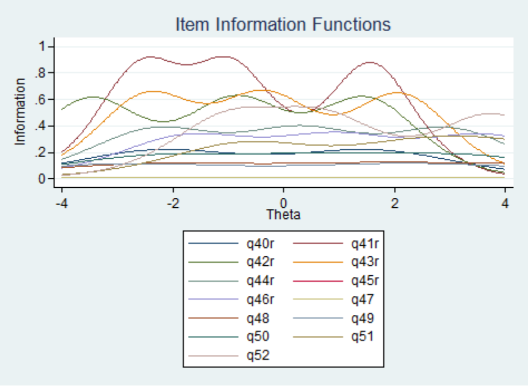

Supplementary Figure S1. Graphical Item Information function results.

**Note - Items numbererd from 4–52 for analysis purposes. These items are preresented as questionnaire items 1-49 within the main manuscript.*
